# Supplementary material for: Arterial effects of anthracycline: structural and inflammatory assessments in non-human primates and lymphoma patients
Source: Clin Sci (Lond). 2025 Jan 15;139(1):29–41. doi: 10.1042/CS20241529 (PMC12203989; doi:10.1042/CS20241529)
Supplement: Supplementary Figure S1 [file CS-139-01-CS20241529-s001.docx]

**SUPPLEMENTAL MATERIAL FOR:**

**Arterial effects of anthracycline: structural & inflammatory assessments in non-human primates and lymphoma patients**

**Short title:** Arterial effects of anthracycline in NHP & humans

Stephen Rankin^1^, Caitlin Fountain^2^ Alastair J Gemmell^3,6^, Daire Quinn^4^, Alasdair Henderson^1^, John McClure^1^, Sandy Small^3,6^, Balaji Venugopal^4,6^, Pamela McKay^4^, Piotr J Slomka^5^, David Colville^1,6^, Mark C Petrie^1^, Giselle C. Meléndez^2^, Ninian N Lang^1^

1. BHF Glasgow Cardiovascular Research Centre, School of Cardiovascular and Metabolic Health, University of Glasgow, Glasgow UK
2. Departments of Internal Medicine, Section on Cardiology and Pathology, Section on Comparative Medicine. Wake Forest University School of Medicine, Winston-Salem, USA
3. Department of Clinical Physics & Bioengineering, NHS Greater Glasgow & Clyde, Glasgow
4. The Beatson West of Scotland Cancer Centre, Glasgow, UK
5. Cedars-Sinai, Division of Artificial Intelligence in Medicine, Department of Medicine, Los Angeles, USA
6. School of Medicine, Dentistry and Nursing, University of Glasgow

**Supplementary Figure 1. Consort diagram.**

**Supplementary table 1. Inter-observer and intra-observer variability**

|  |  | **(95% confidence interval)** | |
| --- | --- | --- | --- |
| **Aortic Segment** | **PET parameter** | **Interobserver agreement** | **Intraobserver agreement** |
| Whole aorta | TBRmax | 0.95 (0.88-0.98) | 0.96 (0.90-0.98) |
|  | TBRmean | 0.97 (0.93-0.99) | 0.97 (0.93-0.99) |
|  | TBR max of active segments | 0.88 (0.64-0.96) | 0.86 (0.39-0.87) |
|  | MDS | 0.94 (0.86-0.98) | 0.90 (0.75-0.96) |
| Ascending | TBRmax | 0.97 (0.93-0.99) | 0.97 (0.92-0.99) |
|  | TBRmean | 0.97 (0.94-0.99) | 0.95 (0.89-0.98) |
|  | TBR max of active segments | 0.79 (0.2-0.96) | 0.88 (0.38-0.98) |
|  | MDS | 0.97 (0.92-0.99) | 0.88 (0.71-0.95) |
| Arch | TBRmax | 0.89 (0.75-0.96) | 0.94 (0.85-0.97) |
|  | TBRmean | 0.95 (0.87-0.98) | 0.95 (0.88-0.98) |
|  | TBR max of active segments | 0.76 (-0.13-0.97) | 0.89 (0.4-0.98) |
|  | MDS | 0.78 (0.52-0.91) | 0.85 (0.66-0.94) |
| Descending | TBRmax | 0.95 (0.88-0.98) | 0.96 (0.91-0.99) |
|  | TBRmean | 0.97 (0.93-0.99) | 0.98 (0.95-0.99) |
|  | TBR max of active segments | 0.85 (0.54-0.96) | 0.87 (0.52-0.97) |
|  | MDS | 0.93 (0.82-0.97) | 0.89 (0.73-0.95) |
| Inter and intra observer agreement was assessed by two-way mixed effect interclass coefficient model in 10% of the cohort (n=10 randomly selected scans) | | | |

**Supplementary Table 2: Comparison of aortic FDG uptake (assessed by the mean TBRmax of the whole aorta) with the presence or absence of cardiovascular risk factors**

|  |  | **Mean TBRmax** | | |  |
| --- | --- | --- | --- | --- | --- |
| **CV risk factor** | **N=** | **Baseline** | **Follow up** | **Difference** | ***p*** |
| **Hypertension** No | 66 | 1.46 | 1.45 | -0.01 | 0.47 |
| Yes | 35 | 1.46 | 1.43 | -0.04 |  |
| **Dyslipidaemia** No | 95 | 1.46 | 1.44 | -0.03 | 0.31 |
| Yes | 6 | 1.43 | 1.46 | 0.04 |  |
| **Ischaemic heart disease** No | 95 | 1.46 | 1.44 | -0.02 | 0.06 |
| Yes | 6 | 1.52 | 1.38 | -0.14 |  |
| **Smoking history** No | 56 | 1.48 | 1.46 | -0.01 | 0.49 |
| Yes | 45 | 1.44 | 1.41 | -0.03 |  |
| **Diabetes** No | 87 | 1.47 | 1.44 | -0.02 | 0.77 |
| Yes | 14 | 1.42 | 1.41 | -0.01 |  |
| **Calcium score** <1000 | 77 | 1.47 | 1.44 | -0.03 | 0.53 |
| ≥1000 | 24 | 1.43 | 1.42 | -0.01 |  |
| **Statin**  No | 75 | 1.46 | 1.45 | -0.01 | 0.26 |
| Yes | 26 | 1.45 | 1.40 | -0.05 |  |
| **BMI** <35 | 91 | 1.46 | 1.44 | -0.02 | 0.6 |
| ≥35 | 10 | 1.46 | 1.41 | -0.05 |  |
| **ESC Baseline CV risk** | | |  |  |  |
| Low risk | 46 | 1.50 | 1.45 | -0.04 | 0.32 |
| Medium risk | 36 | 1.44 | 1.45 | 0.01 |  |
| High/Very high risk | 19 | 1.41 | 1.39 | -0.02 |  |
| Univariate analysis was presumed in 101 patients. Unpaired two-tailed *t* test was performed for univariate analysis with two variable (yes/no) and ANOVA for >2 variables (ESC baseline CV risk). | | | | | |

**Supplementary Table 3: Comparison of aortic FDG uptake (assessed by the mean TBRmax of the whole aorta) by baseline demographics and treatment response**

|  |  |  | **Mean TBRmax** | |  |  |
| --- | --- | --- | --- | --- | --- | --- |
| **Group** | **N=** | **Baseline** | **Follow up** | **Difference** |  | ***p*** |
| **Sex** Male | 54 | 1.46 | 1.42 | -0.05 |  | 0.08 |
| Female | 47 | 1.46 | 1.46 | 0.00 |  |  |
| **Age** ≤70 | 63 | 1.48 | 1.45 | -0.04 |  | 0.19 |
| >70 | 38 | 1.42 | 1.43 | 0.00 |  |  |
| **Cancer stage**  1 | 14 | 1.45 | 1.40 | -0.05 |  | 0.22 |
| 2 | 16 | 1.40 | 1.42 | 0.02 |  |  |
| 3 | 24 | 1.47 | 1.48 | 0.01 |  |  |
| 4 | 47 | 1.48 | 1.43 | -0.05 |  |  |
| **Cumulative dose** | |  |  |  |  |  |
| <250mg/m^2^ | 27 | 1.45 | 1.42 | -0.04 |  | 0.5 |
| ≥250mg/m^2^ | 74 | 1.46 | 1.45 | -0.02 |  |  |
| **Complete response** | |  |  |  |  |  |
| No | 32 | 1.49 | 1.44 | -0.05 |  | 0.17 |
| Yes | 69 | 1.45 | 1.44 | -0.01 |  |  |
| **Deauville score on follow up scan** | | | | | |  |
| 1 | 4 | 1.36 | 1.38 | 0.01 |  | 0.64 |
| 2 | 46 | 1.44 | 1.43 | -0.01 |  |  |
| 3 | 19 | 1.48 | 1.48 | 0.00 |  |  |
| 4 | 11 | 1.46 | 1.43 | -0.03 |  |  |
| 5 | 21 | 1.51 | 1.44 | -0.06 |  |  |
| Univariate analysis was presumed in 101 patients. Unpaired two-tailed *t* test was performed for univariate analysis with two variable (yes/no) and ANOVA for >2 variables (cancer stage & Deauville score). | | | | | | |
